# Supplementary material for: Microplastics in the Lung Tissues Associated with Blood Test Index
Source: Toxics. 2023 Sep 6;11(9):759. doi: 10.3390/toxics11090759 (PMC10534820; doi:10.3390/toxics11090759)
Supplement: Supplementary file 1 [file toxics-11-00759-s001.zip › toxics-2595618-supplementary.pdf]

Title: Microplastics in the lung tissues associated with blood test index

Shuguang Wang[1, 2, #], Wenfeng Lu[1, 2, #], Qingdong Cao[3, #], Changli Tu[4], Chenghui Zhong[2], Lan Qiu[2], Saifeng Li[2], Han Zhang[2], Meiqi Lan[2], Liqiu Qiu[2], Xiaoliang Li[5], Yuewei Liu[6], Yun Zhou[1, 2, *], Jing Liu[4, *]

Author Affiliations:

[1]State Key Laboratory of Respiratory Disease, The First Affiliated Hospital of Guangzhou Medical University, Guangzhou, Guangdong 510120, China

[2]School of Public Health, Guangzhou Medical University, Guangzhou, Guangdong 511436, China

[3]Department of Thoracic Surgery, the Fifth Affiliated Hospital of Sun Yat-sen University, Sun Yat-Sen University, Zhuhai, Guangdong Province 519000, China

[4]Department of Pulmonary and Critical Care Medicine, The Fifth Affiliated Hospital of Sun Yat-sen University, Guangzhou, Guangdong 519000, China

[5]Zhuhai Center for Chronic Disease Control and Prevention, Zhuhai, Guangdong 519060, China

[6]Department of Epidemiology, School of Public Health, Sun Yat-sen University, Guangzhou, Guangdong 510080, China

[#]These authors contribute equally to this work.

***Corresponding Authors:**

Jing Liu, PhD, Department of Pulmonary and Critical Care Medicine, The Fifth Affiliated Hospital of Sun Yat-sen University, 52 Meihua East Road, Zhuhai, Guangdong 519000, China; e-mail, liujing25@sysu.edu.cn.

Yun Zhou, PhD, State Key Laboratory of Respiratory Disease, The First Affiliated Hospital of Guangzhou Medical University, 151 Yanjiang Road, Guangzhou, Guangdong 510120, China;

School of Public Health, Guangzhou Medical University, 1 Xinzao Road, Guangzhou, Guangdong 511436, China; e-mail, yunz@gzhmu.edu.cn.

Supplementary Materials

**Content**	**Page**
**Table S1. Characteristics of MPs exposure in the lung tissue and control samples.**	**3**
**Table S2. Information of each patient.**	**6**
**Table S3. Correlation coefficients of MPs in the lung tissues and blood test index.**	**8**

Table S1. Characteristics of MPs exposure in the lung tissue and control samples.

Sample ID	MPs abundance	Weight of sample (g)	Concentrations (particles/g)[b]	Average Diameter (μm)	Lung tissue portion	20-100 (μm)			100-500 (μm)		
						Type	Number	Fiber[c]	Type	Number	Fiber[c]
1	4	1.96	2.04	60.70	Right superior lobe	PP	2	1			
						PET	2				
2	3	2.49	1.20	86.82	Left inferior lobe	EVA	1				
						PET	1				
									PU	1	
3	32	1.82	17.58	43.88	Left superior lobe	PP	24	12	PP	2	2
						BR	3	1			
						CPE	2				
						ACR	1				
4	4	3.79	1.06	62.69	Right inferior lobe	PET	2				
						PU	1	1			
									PVC	1	
5	15	1.93	7.77	67.79	Left superior lobe	PET	5		PET	1	
						ACR	2				
						PS	2				
						EVA	1		EVA	1	
						PE	1				
						PVC	1				
									PP	1	
6	6	2.04	2.94	30.79	Right inferior lobe	CPE	4				
						ACR	1				
						PET	1				

7	1	1.84	0.54	31.92	Left inferior lobe	PVC	1				
8	3	1.99	1.51	73.83	Left inferior lobe	PP	1				
						PET	1				
						ACR	1				
9	0	1.89	0.00		Left superior lobe						
10	4	1.71	2.34	41.18	Left inferior lobe	PP	2				
						PVC	1				
						SIL	1				
11	16	1.69	9.47	58.80	Left superior lobe	PET	7		PET	1	1
						PS	4				
						PP	3				
						PVC	1				
12	20	3.84	5.21	65.91	Right inferior lobe	PTFE	5		PTFE	2	
						PE	4				
						PS	3	1			
						PET	2				
						PP	1		PP	1	
						PVC	1		PVC	1	
control1	0	5.00	0.00	-		-	-		-	-	
control2	5	5.00	1.00	56.81		PU	3				
						PP	1		PP	1	
control3	2	5.00	0.40	31.11		EPN	1				
						PET	1				

Note: MPs: microplastics, PP: polypropylene, PET: polyethylene terephthalate, PS: polystyrene, PVC: polyvinylchloride, PTFE: polytetrafluoroethylene, CPE: chlorinated polyethylene, PE: polyethylene, ACR: acrylates, EVA: ethylene vinyl acetate, BR: butadiene rubber, PU: polyurethane, SIL: silicone, EPN: phenolic epoxy resin.

[a] MPs with a match degree ≥ 0.80 were included.

[b] The concentrations of MPs (particles/g) were calculated by dividing the quantity of MPs by the weight of the relevant lung tissue sample or control sample.

[c] Fibers were defined as MPs with length-to-diameter ratios ≥ 3.

Table S2. Information of each patient.

Sample ID	1	2	3	4	5	6	7	8	9	10	11	12
**Sex**	Femal e	Male	Fem ale	Male	Female	Male	Male	Male	Male	Male	Fe mal e	Female
**Age, years**	29	55	69	57	49	50	60	67	43	59	64	59
**Wearing of face masks, hours per day**	2.5	9	3.5	1	2.5	12	2	6	9	2.5	5	8
**Educational level**	Junior college	Senior high school	illite racy	Junior college	Junior high school	Senior high school	Senior high school	Primar y school	Junior college	Senior high school	coll ege	Primar y school
**BMI level**	18.80	25.54	25.3 2	25.86	30.28	22.15	27.58	23.21	26.22	22.44	23. 23	25.13
**Seafood consumption, times per week**	4~6	1~3	1~3	4~6	1~3	1~3	7	1~3	4~6	1~3	<1	7
**Traffic pollution exposure time, minutes per day**	30~60	30~60	<10	30~60	10~30	60~120	<10	10~30	<10	<10	<10	<10
**Distance between residence and nearest major roads, meter**	500~1 000	300~500	≥10 00	≥1000	50~100	50~100	300~500	≥1000	300~5 00	100~300	50~ 100	100~30 0
**Working indoors**	Y	Y	N	Y	Y	Y	Y	Y	Y	Y	Y	N

**(Y/N)**												
**Alcohol consumption (Y/N)**	N	Y	N	N	N	N	N	N	N	N	Y	N
**Self-cooking (Y/N)**	Y	Y	Y	N	N	N	N	Y	N	Y	N	Y
**Hypertension (Y/N)**	N	N	Y	N	N	N	Y	N	N	N	N	Y
**Diabetes (Y/N)**	N	N	N	N	N	N	N	N	N	N	N	Y
**Coronary heart disease (Y/N)**	N	N	N	N	N	N	N	N	N	N	N	N
**Lung or upper-airway surgeries (Y/N)**	N	N	N	N	N	N	N	N	N	N	N	N

Table S3. Correlation coefficients of MPs in the lung tissues and blood test index.

Blood test index	Correlation coefficient	*p* -value[b]
WBC ($\times10^9$/L)	0.32	0.31
RBC ($\times10^9$/L)	-0.57	0.06
HGB (g/L)	-0.57	0.05
PLT ($\times10^9$/L)	0.78	< 0.01
Hematocrit (%)	-0.57	0.05
MCV (fL)	0.15	0.64
Mean red cell hemoglobin content (pg)	-0.25	0.43
MCHC (g/L)	-0.35	0.26
RDW SD (fL)	0.27	0.39
RDW CV (%)	< 0.01	0.99
N ($\times10^9$/L)	0.20	0.53
L ($\times10^9$/L)	0.04	0.90
N / L	0.08	0.81
MON# ($\times10^9$/L)	-0.03	0.93
EOS# ($\times10^9$/L)	0.36	0.26
Thrombocytocrit (%)	0.82	< 0.01
PDW (fL)	-0.18	0.57
MPV (fL)	-0.20	0.53
PT (S)	0.01	0.98
APTT (S)	-0.13	0.69
FIB (g/L)	0.63	0.03
Urea (mmol/L)	-0.41	0.19
Cr (μmol/L)	-0.40	0.19
UA (μmol/L)	-0.38	0.23
K^+ (mmol/L)	-0.15	0.64
Na^+ (mmol/L)	0.48	0.11
Cl^- (mmol/L)	0.14	0.67
GLU (mmol/L)	< 0.01	1.00
AST (U / L)	-0.43	0.17
ALT (U / L)	-0.11	0.73
TB (μmol/L)	-0.66	0.02
DB (μmol/L)	-0.78	< 0.01
Hemobilirubin (μmol/L)	-0.62	0.03
TP (g/L)	0.22	0.48
Albumin (g/L)	-0.06	0.86
Globulin (g/L)	0.38	0.23

Note: MPs: microplastics, WBC: white blood cell, RBC: red blood cell, HGB: hemoglobin, PLT: platelet, MCV: mean corpuscular volume, MCHC: mean corpuscular-hemoglobin concentration, RDW: red blood cell volume distribution width, N: neutrophil, L: lymphocyte, MON#: monocyte, EOS#: eosinophil, PDW: platelet distribution width, MPV: mean platelet volume, PT: prothrombin time, APTT: activated partial thromboplastin time, FIB: fibrinogen, Cr: Creatinine, UA: blood uric acid, GLU: glucose, AST: aspartate aminotransferase, ALT:

alanine aminotransferase , TB: total bilirubin, DB: direct bilirubin, TP: total protein.

[a] MPs with a match degree ≥ 0.80 were included.

[b] The p -value was estimated using the Mann−Whitney U test.
